# Green Production of Zero-Valent Iron (ZVI) Using Tea-Leaf Extracts for Fenton Degradation of Mixed Rhodamine B and Methyl Orange Dyes

**DOI:** 10.3390/ma15010332

**Published:** 2022-01-03

**Authors:** Diana Rakhmawaty Eddy, Dian Nursyamsiah, Muhamad Diki Permana, Solihudin, Atiek Rostika Noviyanti, Iman Rahayu

**Affiliations:** Department of Chemistry, Faculty of Mathematics and Sciences, Universitas Padjadjaran, Jl. Raya Bandung-Sumedang Km. 21 Jatinangor, Sumedang 45363, Indonesia; diannursyamsiah@gmail.com (D.N.); muhamad16046@mail.unpad.ac.id (M.D.P.); solihudin@unpad.ac.id (S.); atiek.noviyanti@unpad.ac.id (A.R.N.); iman.rahayu@unpad.ac.id (I.R.)

**Keywords:** dye degradation, Fenton method, green synthesis, zero-valent iron

## Abstract

The danger from the content of dyes produced by textile-industry waste can cause environmental degradation when not appropriately treated. However, existing waste-treatment methods have not been effective in degrading dyes in textile waste. Zero-valent iron (ZVI), which has been widely used for wastewater treatment, needs to be developed to acquire effective green production. Tea (*Camellia sinensis*) leaves contain many polyphenolic compounds used as natural reducing agents. Therefore, this study aims to synthesize ZVI using biological reducing agents from tea-leaf extract and apply the Fenton method to degrade the color mixture of rhodamine B and methyl orange. The results show that the highest polyphenols were obtained from tea extract by heating to 90 °C for 80 min. Furthermore, PSA results show that ZVI had a homogeneous size of iron and tea extract at a volume ratio of 1:3. The SEM-EDS results show that all samples had agglomerated particles. The ZVI 1:1 showed the best results, with a 100% decrease in the color intensity of the dye mixture for 60 min of reaction and a degradation percentage of 100% and 66.47% for rhodamine B and methyl orange from LC-MS analysis, respectively. Finally, the decrease in COD value by ZVI was 92.11%, higher than the 47.36% decrease obtained using Fe(II).

## 1. Introduction

The textile industry is one of the fastest-growing sectors in Indonesia, and according to data from the Indonesian Ministry of Industry, it grew by 15.08% in 2019 [1]. The number of textile industry activities is directly proportional to the amount of wastewater generated. Dyestuff waste produced is toxic because it contains carcinogenic aromatic amines [2,3]. When waste is not appropriately treated, it impacts environmental degradation and disrupts public health. Therefore, effective and environmentally friendly treatment of industrial wastewater, especially dye waste, is needed. The commonly used methods in wastewater treatment to reduce dye content include adsorption, coagulation, and flocculation methods [4,5,6,7]. However, using these methods to minimize dyestuffs is often not perfect because it requires a large amount of coagulant. Other methods used in wastewater treatment are the membrane technique and bacterial biodegradation [8,9]. However, the regeneration process is complex, the cost is high, and the sludge generated from treatment residue is significant [10,11,12]. Therefore, modifications to existing water-treatment methods are needed.

Zero-valent iron (ZVI) was initially used for groundwater remediation. However, along with increasingly complex surface-water problems, ZVI can be applied to surface-water and wastewater treatment to reduce the number of pollutants [13,14,15,16]. Zero-valent iron material is made by reducing iron(II) or iron(III) using sodium borohydride [17,18,19]. Sodium borohydride is considered less environmentally friendly as a reducing agent. Therefore, several studies have developed a synthesis method that is more environmentally friendly. Reducing agents that have been widely used are plant extracts. Subsequently, plants with polyphenol content and high antioxidant capacities, such as strawberries, raspberries, black tea, eucalyptus, mulberry, green tea, pomegranate, and oak leaves, can act as a reducing agent in the production of ZVI [20,21,22,23,24,25,26]. Tea leaves (*Camellia sinensis*) are one of the most abundant commodities, with polyphenols as the essential content in the form of flavonoids and catechins ranging from 20 to 30% [27,28]. In 2013, production capacity reached 152,700 tons/year [29]; therefore, tea-leaf extract can be used as an iron-reducing agent.

The performance of ZVI in degrading organic pollutants can be improved when applied to the Fenton method. This method is widely studied because it is considered effective in reducing organic pollutants. It is simple, can be carried out at ambient temperature and pressure, is easy to handle, and is safe for the environment [30]. The Fenton method is an advanced oxidation process (AOPs) system that works by utilizing hydroxyl radicals (•OH) to degrade pollutants [31]. ZVI using this method consists of iron(II) as a catalyst and hydrogen peroxide as an oxidizing agent [32,33]. Several studies have reported that ZVI applied to the Fenton method is effective in reducing the color intensity of bromothymol blue, methylene blue, a mixture of Remazol brilliant blue R (RBB-R) and direct red (DR), Eriochrome blue-black B (EBB), and malachite green [34,35,36,37].

The dyes used in the textile dyeing process are classified into several types, including nitro, azo, diphenylmethane, triphenylmethane, xanthene, phthalein, indigo, thioindigo, and anthraquinone dyes [2]. Rhodamine B is one of the xanthene classes often used in the textile dyeing industry because it is cheap and easy to obtain [38]. Subsequently, azo dyes are widely used since about 60–70% of organic dyes produced globally are members of the azo group [39]. One of the azo dyes, one of the most easily found is methyl orange, which is soluble in water. Several studies have shown that the Fenton method can degrade rhodamine B and methyl orange into simpler intermediates [40,41,42,43,44]. However, no study has degraded a mixture of rhodamine B and methyl orange dyes using the Fenton method with ZVI as a catalyst.

This study synthesized the ZVI catalyst leaf extract from tea (*Camellia sinensis*) as a natural reducing agent. Furthermore, the synthesized ZVI was characterized using particle size analysis (PSA), UV-Vis spectrophotometry, Fourier-transform infrared spectroscopy (FTIR), and electron microscopy. The Fenton method was then tested to degrade a rhodamine B-methyl orange dye mixture. In wastewater treatment, the decrease in the value of chemical oxygen demand (COD) should be considered to analyze the effectiveness and quality of the treated water. The COD value can indicate the level of water pollution by organic pollutants [45].

## 2. Materials and Methods

### 2.1. Materials

The materials used were iron(II) sulfate heptahydrate (FeSO_4_·7H_2_O, 99.5%, Merck 103965, Kenilworth, NJ, USA,), Folin Ciocalteu reagent, which is a mixture of phosphomolybdate and phosphotungstate (2 mol/L, Merck 109001, Kenilworth, NJ, USA), sodium carbonate (Na_2_CO_3_, 99.9%, Merck 106392, Kenilworth, NJ, USA), gallic acid (C_7_H_6_O_5_, 98%, Merck 842649, Kenilworth, NJ, USA), tea leaves (*Camellia sinensis*) from the Citengah tea plantation, Sumedang, Indonesia (Figure S1), concentrated sulfuric acid (H_2_SO_4_, 95–97%, Smart Lab A-1092 F), hydrogen peroxide (H_2_O_2_, 30%, Merck 107209, Kenilworth, NJ, USA), digestion solution for COD (prepared from mercury(II) sulfate (HgSO_4_, 98%, Merck 104480, Kenilworth, NJ, USA) and potassium dichromate (K_2_Cr_2_O_7_, 99,9%, Merck 104864, Kenilworth, NJ, USA), sodium hydroxide (NaOH, 99%, Merck 1006498, Kenilworth, NJ, USA), ammonium iron(II) sulfate hexahydrate ((NH_4_)_2_Fe(SO_4_)_2_·6H_2_O, 99%, Merck 103792, Kenilworth, NJ, USA), ferroin indicator (prepared from 1,10-phenanthroline monohydrate (C_12_H_8_N_2_·H_2_O, 99,5%, Merck 107225, Kenilworth, NJ, USA) and iron(II) sulfate heptahydrate (FeSO_4_·7H_2_O, 99.5%, Merck 103965, Kenilworth, NJ, USA)), rhodamine B (C_28_H_31_ClN_2_O_3_, 90%, Merck 107599, Kenilworth, NJ, USA), and methyl orange (C_14_H_14_N_3_NaO_3_S, 85%, Merck 114510, Kenilworth, NJ, USA).

### 2.2. Tea Extract Preparation

Tea leaves (*Camellia sinensis*) were taken from the Citengah tea plantation, Sumedang (Figure S1), and dried until the water was gone [20]. The leaves were then separated from the stems and cut into small pieces to an area of 1 × 1 cm. Furthermore, they were heated at 50 °C for 48 h and at 105 °C for 4 h to determine water content. Finally, the dried tea leaves were ground and then sieved through of 10 mesh.

### 2.3. Determination of Polyphenol Content in Tea Extract

The tea extract was made by modifying the existing procedures in previous studies [25,46]. First, 3.6 g of tea powder of was added to 100 mL of distilled water as a solvent, then heated at various temperatures of 28–98 °C for 20 min, filtered and concentrated. A total of 10 mg of extract was then dissolved with 50 mL of distilled water. Then, 5 mL of the extract was taken, 0.2 mL of Folin Ciocalteu reagent, 4 mL of 7.5% sodium carbonate was added, and distilled water was added to a volume of 25 mL. The solution was incubated for 45 min at room temperature and measured using a visible spectrophotometer (HACH DR 3900, Loveland, CO, USA) at a wavelength of 698 nm. Then, the optimum heating time was found, with time variations of 20–100 min using the same method. Finally, the total polyphenol content was calculated using Equation (1).

TPC = (C × V)/m(1)

where TPC is the total phenolic content (mg GAE/g), C is the concentration of tea leaves (mg/L), V represents the volume of solvent (L), and m is the weight of the tea extract used (g).

### 2.4. Synthesis and Characterization of Zero-Valent Iron (ZVI)

The synthesis of ZVI was carried out using a modified method from a previous study [25]. First, the tea extract was added with 0.1 mol/L of iron(II) sulfate solution with a volume ratio of iron(II) to tea extract of 1:1, 1:2, and 1:3 while stirring in a sonicator-900W (BEM-900A, Bueno Biotech) in a stream of nitrogen gas. The remaining water was then evaporated, and ZVI was obtained.

The synthesized ZVI was characterized using a UV-Vis spectrophotometer (Thermo Fisher Scientific Genesys 10S, Waltham, MA, USA) to determine the absorption at a 200–600 nm wavelength. ZVI was further described using Fourier-transform infrared spectroscopy (FTIR, Shimadzu IRPrestige-21, Tokyo, Japan) to determine the functional group, while FTIR was used, with a scanning range of 400–4000 cm^−1^. Scanning electron microscope-energy-dispersive X-ray spectrometry analysis (SEM-EDS, Hitachi SU3500, Tokyo, Japan) was performed using 3 kV at 2000× magnification to determine the shape of the surface morphology. Finally, particle size was determined using a particle-size analyzer (PSA, Horiba SZ-100, Kyoto, Japan).

### 2.5. ZVI Catalytic Test for Dyes

Samples of a mixture of rhodamine B and methyl orange, 1:1 (each 50 mg/L), were tested for color intensity. First, the sample was adjusted to pH 2, then, 5 mL of 12% hydrogen peroxide and ZVI with various concentrations of 50, 100, and 150 mg/L. Next, the solution was stirred, and the intensity of the color was measured using a UV-Vis spectrophotometer (Thermo Fisher Scientific Genesys 10S, Waltham, MA, USA) at a wavelength of 200–600 nm. The measurement process was carried out for 180 min with an interval of 30 min. Meanwhile, to determine the presence of degradation and intermediate compounds formed, testing was carried out using liquid chromatography-mass spectrometry (LC-MS, Waters Q-tof MS Xevo, Milford, MA, USA).

### 2.6. Chemical Oxygen Demand (COD) Test

The COD measurement in the sample refers to the measurement of water using the closed reflux method. First, a total of 2.5 mL of sample was put into a reflux tube. Then, 1.5 mL of digestion solution and 3.5 mL of concentrated sulfuric acid were added. The tube was inserted into the COD reactor (Velp ECO 25, Usmate Velate, Italy) and heated at 165 °C for 2 h. After cooling, the sample was titrated using a standard solution of 0.1 mol ek/L ammonium iron(II) sulfate with ferroin as an indicator.

(2) $$\mathrm{COD}=\frac{\left(V_{1-}V_{2}\right)\times {\;C}_{\mathrm{FAC}}\times 8\times 1000\;}{V_{3}}$$

where chemical oxygen demand (COD) is represented in mg O_2_/g, C_FAC_ is the concentration of ammonium iron(II) sulfate (mol ek/L), V_1_ is the blank titration volume (mL), V_2_ is the sample titration volume (mL), and V_3_ is the sample volume (mL).

## 3. Results and Discussions

### 3.1. Preparation and Polyphenol Content of Tea Extract

**Figure 1:**
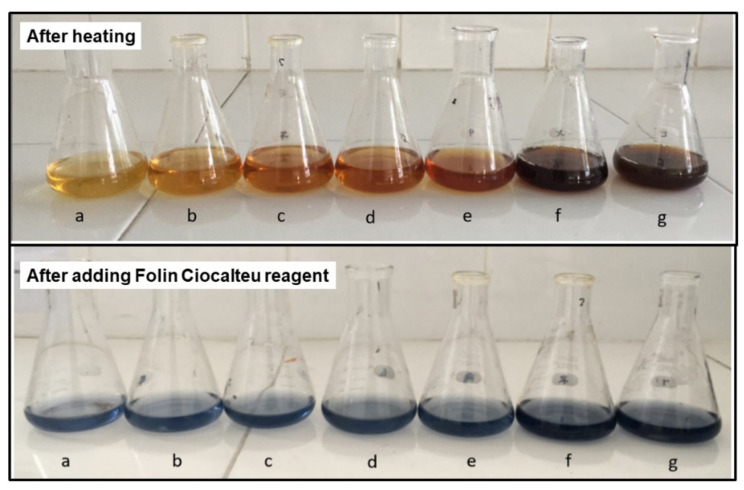
Tea extract after heating for 20 min and after the addition of Folin Ciocalteu reagent at: (**a**) 28; (**b**) 50; (**c**) 60; (**d**) 70; (**e**) 80; (**f**) 90; and (**g**) 98 °C.

The extract was prepared using the shoots to leaves without stems, which had a moisture content of 63.43 ± 0.50%. Polyphenols in catechins found in tea leaves were polar compounds extracted with water [47]. The experimental results in Figure 1 show that the higher the heating temperature used, the more concentrated the color of the extract obtained; therefore, more polyphenolic compounds were extracted. The amount of polyphenol content in the extract can be confirmed by the Folin Ciocalteu method. Besides polyphenols, several water-soluble compounds, such as caffeine, amino acids, and sugars in tea, can also be extracted. As a result, the compound components contained in the extract are not in the form of a single compound [48,49].

**Figure 2:**
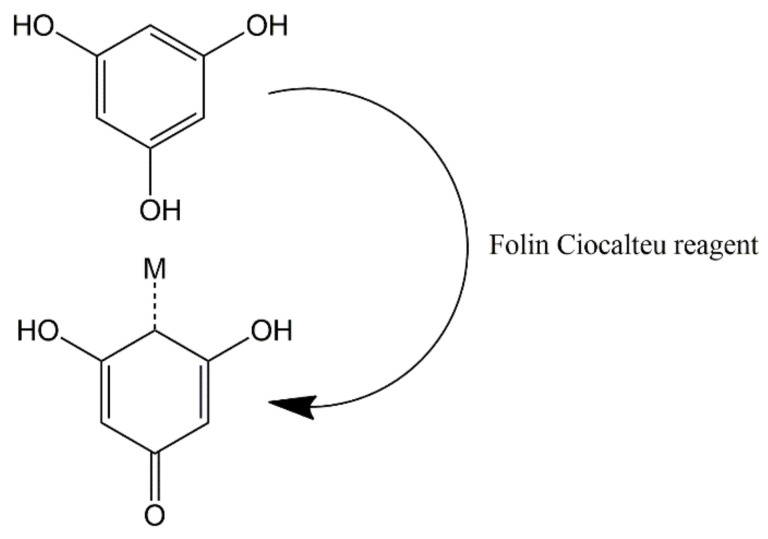
Redox reaction between polyphenols and Folin Ciocalteu reagent.

The polyphenol content in tea extract was determined using the Folin Ciocalteu method. Folin Ciocalteu reagent is a complex compound formed from phosphomolybdic acid and heteropoly phosphotungstic acid. The working principle is the oxidation of hydroxyl groups in phenolic compounds by Folin Ciocalteu reagent. Furthermore, the blue color of the solution is the reduced form of the Folin Ciocalteu reagent [50,51]. Gallic acid is used as a standard in the determination of polyphenol content. The maximum wavelength of standard gallic acid and the optimal incubation time are shown in Figure S2 and the calibration curve for gallic acid shown in Figure S3. Polyphenol measurement data showed that the more concentrated the resulting color (Figure 1), the higher the polyphenol content in the extract. Figure 2 shows the redox reaction between polyphenols and the Folin Ciocalteu reagent [51]. 

**Figure 3:**
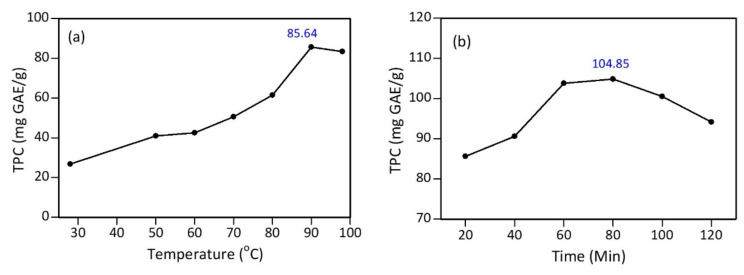
Total polyphenol content (TPC) of tea extract: (**a**) variation of temperature with 20 min heating; and (**b**) variation of heating time at 90 °C.

The test results found that the maximum total phenolic content (TPC) was obtained at a temperature of 90 °C, with a TPC value of 85.64 mg GAE/g (Figure 3a). Furthermore, several time variations were tested using the optimum temperature of 90 °C. The data showed that the polyphenol content of the tea extract heated at 90 °C for 80 min increased by 18.35% to 104.85 mg GAE/g (Figure 3b), compared to heating for 20 min. Therefore, based on these results, the optimum extraction process was conducted at 90 °C for 80 min.

### 3.2. Characteristics of ZVI

**Figure 4:**
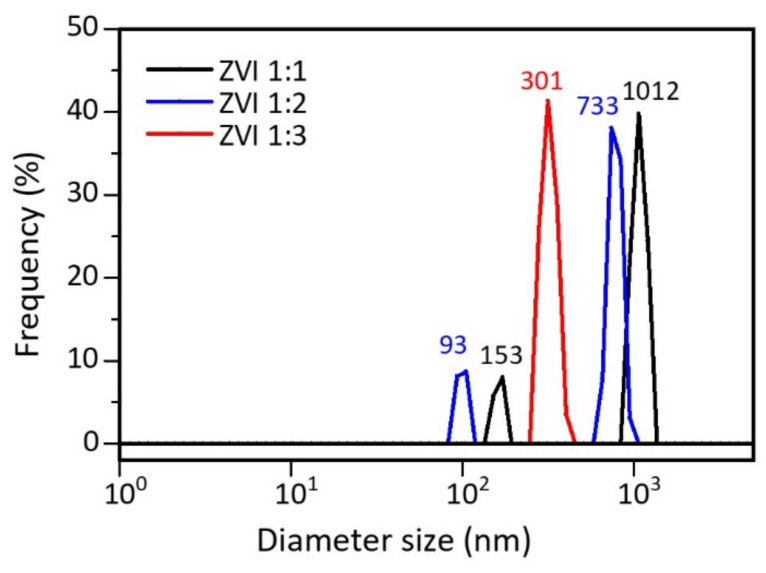
PSA results for ZVI particle size distribution.

The particle-size distribution of ZVI was tested using a particle-size analyzer (PSA). Table 1 presents data that shows that ZVI 1:1 and 1:2 are distributed in two sizes, indicated by the presence of two peaks formed, while ZVI 1:3 shows one peak (Figure 4). Therefore, the more tea extract used, the smaller and more uniform the particle size. This is understandable because the polyphenols in tea extract act as reducing and capping agents to prevent the agglomeration process [52]. The presence of hydrophobic poles on the capping agent causes the formation of steric barriers that can control particle growth. It reduces the surface energy of the particles, and aggregation can be avoided [53,54]. Apart from polyphenols, several compounds that are naturally contained in plants, such as citric acid, vitamins, and silica, can also act as natural capping agents [55].

**Figure 5:**
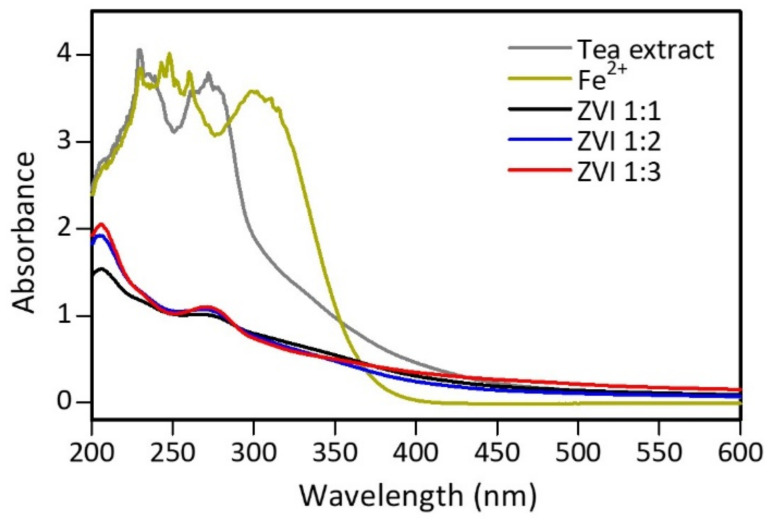
UV-Vis spectra of tea extract, 0.1 M iron(II), and ZVI.

UV-Vis spectrum analysis was conducted to analyze the difference in peaks produced by tea extract, iron(II) solution, and ZVI produced from the synthesis process, and the measurement was carried out at a 200–600 nm wavelength [56]. Figure S4 shows the color change of the iron(II) solution on the formation of nZVI. The test results show differences in absorption peaks, indicating that a new product, which is different from the previous constituent materials, was formed (Figure 5). The tea extract has peaks at wavelengths of 230 and 270 nm, indicating the presence of phenolic acid and its derivatives [23]. Meanwhile, the iron(II) solution showed absorption peaks in the 230–260 and 300 nm regions, probably from [FeHSO_4_]^2+^ [57]. In the ZVI spectrum, the resulting peak of the [FeHSO_4_]^2+^ ion did not appear, indicating that the compound had changed. However, there was a peak at 270 nm at various iron(II) ratios and tea extract but less intense than before. This peak indicates that phenolic compounds and their derivatives are still present in the ZVI material. Around 200 nm, a typical peak of benzoyl in flavonoid compounds is shown from tea extract [58]. Other compounds besides polyphenols identified in ZVI were present since the extract used was not pure isolate. Furthermore, experimental data also showed that the more tea extract used, the higher the absorption peaks at wavelengths of 200 and 270 nm. This occurred because the number of polyphenols and compounds added to the extract increased, while the iron(II) remained. Therefore, there was an excess of unreacted polyphenolic compounds.

**Figure 6:**
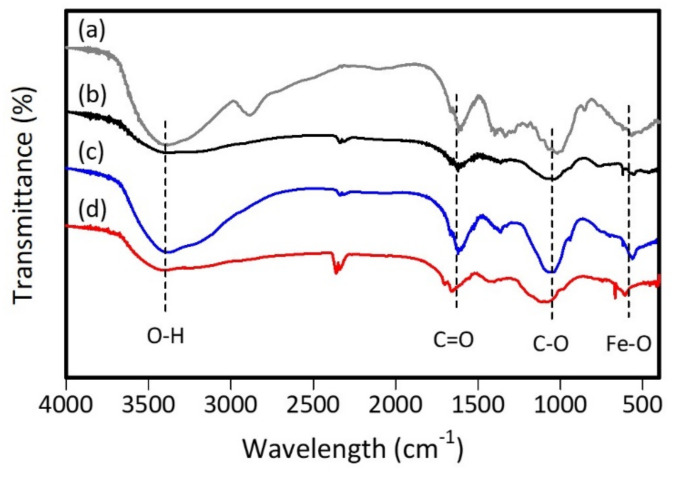
FTIR spectra of (**a**) tea extract, (**b**) ZVI 1:1, (**c**) ZVI 1:2, and (**d**) ZVI 1:3.

FTIR measurements determine the functional groups in tea extract and ZVI. Figure 6 shows the absorption band at wavenumber 3392–3407 cm^−1^ from O–H stretching vibrations of polyphenol compounds [59,60]. Meanwhile, the absorption band at wavenumber 1663–1655 cm^−1^ and 1450–1500 cm^−1^ comes from the C=O vibration and C=C vibration of the aromatic ring [61]. Then, the absorption band at wavenumber 1064–1099 cm^−1^ comes from the C–O bond of the heterocyclic pyranose ring [62].

The sp^3^ carbon signal appears in the tea extract spectrum at a wavenumber of 2894 cm^−1^; this signal can come from lignin dissolved during the extraction process. The C–H stretch signal of aromatic methoxy and methylene groups that are characteristic of lignin are shown at wavenumber 2851–2924 cm^−1^ [63]. However, this signal disappeared in the ZVI spectrum due to the widening of the hydroxyl group band and formation of bonds between iron and hydroxyl groups of polyphenol compounds [64]. In the ZVI spectrum, a signal appears at wavenumber 2354–2363 cm^−1^ from the saturated bond of carbon dioxide [65]. Meanwhile, the absorption band at wavenumber 606–610 cm^−1^ comes from the Fe–O vibration [66].

**Figure 7:**
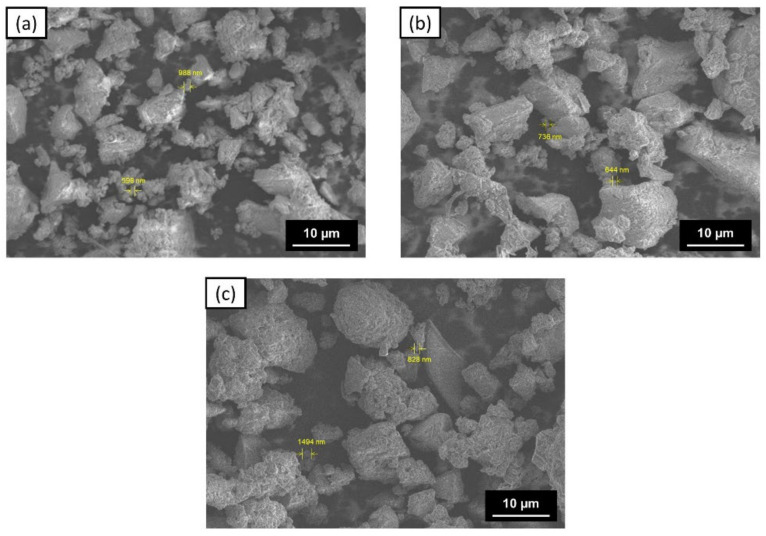
SEM images of: (**a**) ZVI 1:1; (**b**) ZVI 1:2; and (**c**) ZVI 1:3.

The morphology of the ZVI surface was determined using SEM-EDS, and the various shapes and sizes indicated the presence of agglomeration in the three types of ZVI (Figure 7). Agglomeration can occur due to the formation of an organic layer of tea extract and iron oxide on the surface of ZVI [58,59]. This is supported by the results of FTIR measurements, which showed that there are still several functional groups detected on the surface of ZVI. Moreover, the presence of soluble fibers, such as lignin, which was confirmed in the FTIR measurement, can also cause the outer layer of ZVI to be thicker [67].

EDS measurement results show that the main components on the surface of ZVI are carbon, oxygen, sulfur, and iron (Table 2). However, the iron element on the surface is not the primary constituent because there may be an agglomeration of the organic layer that dominates the ZVI surface. Meanwhile, the sulfur in ZVI can come from ferrous sulfate, the precursor of ZVI. Other sodium, nitrogen, and potassium elements may have come from tea extract, which is not a pure isolate [25,68]. Based on the analysis results, the more tea extract used, the more carbon and oxygen measured on the material’s surface. This further confirms and supports an organic layer on the ZVI surface. Meanwhile, the estimated iron content was not significantly different.

### 3.3. Color Intensity Test

**Figure 8:**
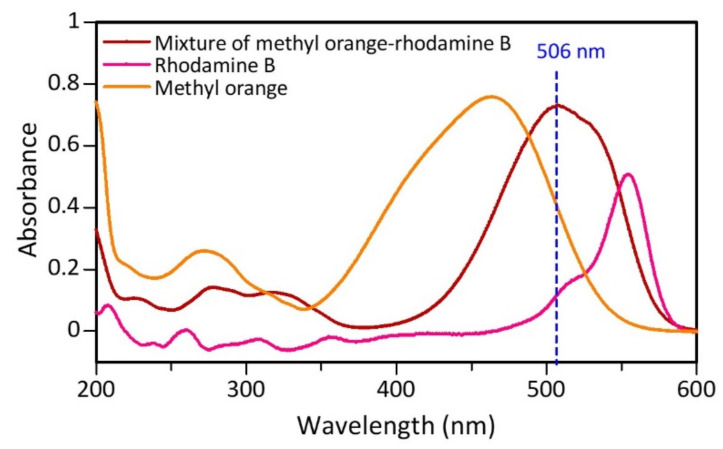
Maximum wavelength determination curve before the catalytic process for the dyes methyl orange, rhodamine B, and mixture (methyl orange-rhodamine B).

The dye sample used was a mixture of rhodamine B and methyl orange in a ratio of 1:1 with a concentration of 50 mg/L, respectively. Samples of mixed dyestuffs that have not been catalyzed are determined by their maximum wavelength using UV-Vis. From the test results, each component of rhodamine B, methyl orange, and mixture (methyl orange-rhodamine B) has a maximum wavelength of 555, 464, and 506 nm, respectively (Figure 8). The maximum wavelength of the mixture shifted from each of its constituent components, indicating that there was a change in the chromophore structure due to the interaction between rhodamine B and methyl orange.

**Figure 9:**
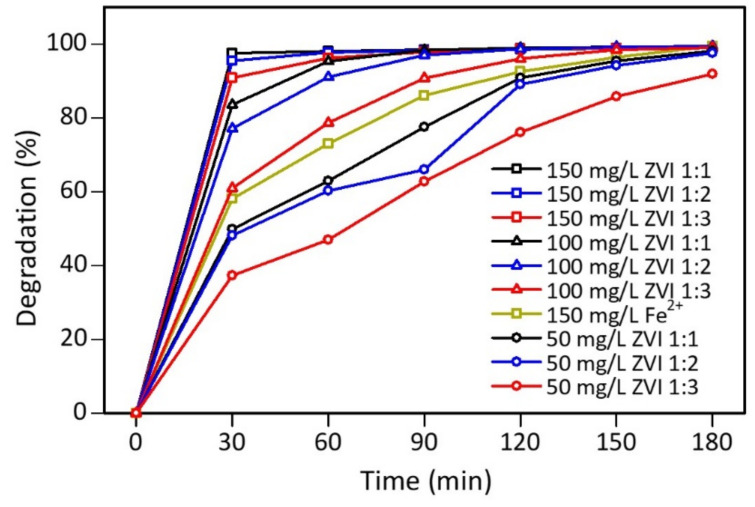
Decreased color intensity of mixture of rhodamine B-methyl orange at 506 nm.

Figure 9 shows that ZVI 1:1 obtained the most significant decrease in color intensity, by 49.82%, followed by ZVI 1:2 and ZVI 1:3, by 48.12% and 37.28%, respectively, after 30 min of contact. This happened because ZVI 1:3 has the highest carbon and oxygen content on its surface based on the characterization results. Furthermore, on UV-Vis measurement, the peak of flavonoids and phenolics was most significant at ZVI 1:3 compared to other types of ZVI. Therefore, the presence of these compounds can interfere with the performance of iron(0) in the core of the material. Even though the PSA results of ZVI 1:3 showed the most uniform size, the catalytic process of the Fenton method was influenced by other factors, such as surface composition. Therefore, the particle size of the iron catalyst did not significantly affect the performance [69].

Figure 9 also signifies that the greater the concentration of ZVI used in the catalytic process, the more significant the decrease in the color intensity of the mixture. Furthermore, contact time also affects the decrease in the color intensity of the mixture, where the longer the contact process, the less the color intensity of the mixture. ZVI 1:1, 1:2, and 1:3 catalysts with a concentration of 50 mg/L reduced the dye up to >90% when the contact time reached 120 min. Meanwhile, at a concentration of ZVI 100 mg/L, the color intensity decreased >90% when the Fenton process lasted for 60 min. At ZVI 150 mg/L, the color intensity decreased to 90% only after a contact time of 30 min. Iron(II) sulfate 150 mg/L within 30 min reduced the color intensity of the mixture by 58.07%; therefore, ZVI had 32% more effective ability.

An analysis was also carried out to see any changes in the peak at a wavelength of 200–600 nm during the Fenton process. The results showed that the mixed dyes that underwent a catalytic process using ZVI 1:1, 1:2, and 1:3 with concentrations of 50, 100, and 150 mg/L, respectively, underwent the same changes during the catalytic process from 30–180 min (Figures S5–S8). The peak at the wavelength of 506 nm did not shift and only experienced a decrease in color intensity, while the peak around the wavelength of 200–300 nm experienced a change. This peak is thought to originate from ZVI that has been converted to iron(II) during the Fenton process.

**Figure 10:**
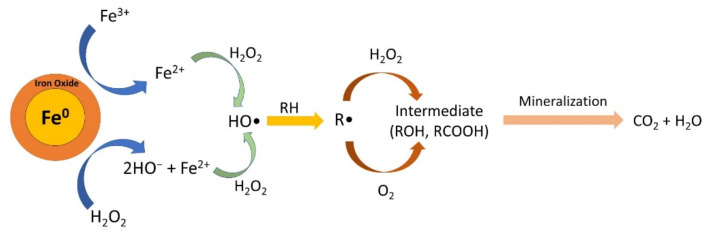
Mechanism of heterogeneous Fenton reactions with ZVI.

The mechanism of Fenton’s reaction with ZVI is illustrated in Figure 10. Fe^0^ is oxidized by H_2_O_2_ to give Fe^2+^ in Equation (3). In addition, Fe^0^ can also reduce Fe^3+^ to Fe^2+^ in Equation (4). Then, Fe^2+^ will react with H_2_O_2_ to form a highly reactive hydroxyl radical (•OH) (Equation (5)). These hydroxyl radicals then degrade organic pollutants so that they are oxidized, which is written in Equations (6)–(8), which, in the end mineralization, occurs to form CO_2_ and H_2_O end products.

Fe^0^ + H_2_O_2_ → Fe^2+^ + 2OH^−^ (3)

2Fe^3+^ + Fe^0^ → 3Fe^2+^ (4)

Fe^2+^ + H_2_O_2_ → Fe^3+^ + HO^−^ + HO• (5)

RH + HO• → H_2_O + R• (6)

R• + H_2_O_2_ → ROH + HO• (7)

R• + O_2_ → ROO• (8)

### 3.4. Degradation Pathway and Identification of the Intermediates

**Figure 11:**
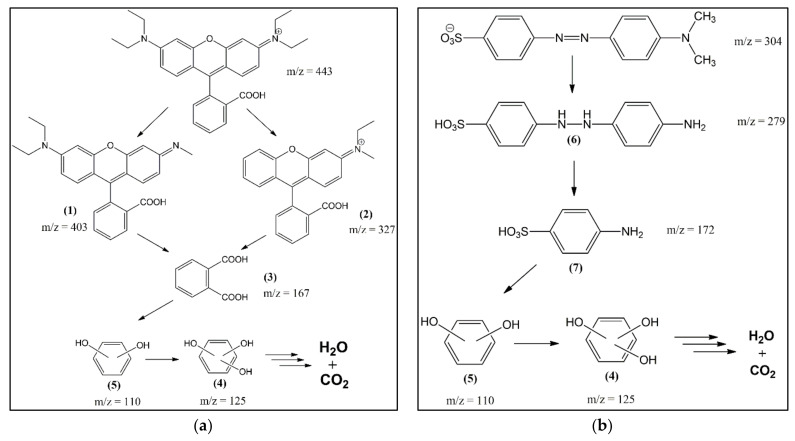
Schematic illustration of the proposed degradation pathway of: (**a**) rhodamine B; and (**b**) methyl orange.

Analysis of reaction intermediates and final products is useful for evaluating the efficiency of a catalytic system and can reveal some details of the reaction process. This study used liquid chromatography-mass spectrometry (LC-MS, Waters Q-tof MS Xevo, Milford, MA, USA) to determine the dye-mixture intermediates. The LC-MS chromatogram results showed that the structure of the rhodamine B fragment before the catalytic process was identified to experience chloride-loss ionization with *m*/*z* 443. Meanwhile, methyl orange before the catalytic process was identified at *m*/*z* 327. A signal appeared with *m*/*z* 304 and 306 from methyl orange, which lost sodium ions and underwent rearrangement [70]. The results of a mixture of dyes refer to the retention time of each component before being mixed. The intermediates of rhodamine B and methyl orange are shown in Table 3. The intact structure of rhodamine B did not reappear on the chromatogram after the catalytic process. This indicates that the dye had degraded entirely to another compound with a smaller molecular mass. Meanwhile, the intact structure of methyl orange with *m*/*z* 304 and 327 still exists. This proves that the catalytic process to degrade methyl orange requires more difficulty than rhodamine B. The proposed mechanism from the degradation of rhodamine B and methyl orange using ZVI with the Fenton method is shown in Figure 11.

Furthermore, using Origin85 8.5.1 SR_2_ software, the area of methyl orange and rhodamine B mixture was measured before and after the catalytic process to determine the degradation level. The peaks selected in the determination of the area were peaks belonging to rhodamine B and methyl orange. Based on the calculation results, rhodamine B and methyl orange areas decreased by 100% and 66.47%, respectively.

### 3.5. COD Testing

In water treatment, especially wastewater, the chemical oxygen demand (COD) value is one of the essential parameters because it shows the pollution level in water by organic compounds. Therefore, the COD value is directly proportional to the pollution level. Based on the experimental results, the value of the mixture dye before the catalytic process was 92.76 mg O_2_/g and decreased by 92.11% to 7.32 mg O_2_/g after the catalytic process (Table 4). Meanwhile, iron(II) as a catalyst only reduced the dye by 44.75%.

## 4. Conclusions

In this research, ZVI was synthesized using a natural reducing agent from tea-leaf extract and applied in the Fenton method to degrade the color mixture of rhodamine B and methyl orange. The results show that the optimum dose of ZVI to reduce the color intensity of the rhodamine B and methyl orange mixture was ZVI 1:1 150 mg/L. Furthermore, the LC-MS test showed a degradation process for the rhodamine B and methyl orange mixed dyes, where rhodamine B and methyl orange were degraded by 100% and 66.47%, respectively. Meanwhile, the decrease in the COD value obtained in this condition was 92.11%, which is higher than using Fe(II) of 47.36%.

## Figures and Tables

**Table 1:** Size distribution of ZVI particles.

ZVI	Size Range (nm)		Average Size (nm)	
	First Peak	Second Peak	First Peak	Second Peak
1:1	151–171	945–1207	153	1012
1:2	93–105	740–945	93	733
1:3	279–356	-	301	-

**Table 2:** Weight percent of EDS measurement results for ZVI 1:1, 1:2, and 1:3.

Element		Weight Percent (%)	
	ZVI 1:1	ZVI 1:2	ZVI 1:3
O	36.97	42.92	44.91
C	19.97	20.96	27.98
S	13.39	14.46	5.40
Fe	10.12	11.30	11.90
Na	3.42	2.69	3.24
K	3.32	4.17	2.25
N	2.97	3.50	4.32

**Table 3:** Main intermediates from the degradation of rhodamine B and methyl orange detected by LC-MS.

Compound No.	Retention Time (min)	*m*/*z*
(1)	12.02	403
(2)	10.32	327
(3)	13.27	167
(4)	0.87	125
(5)	24.13	110
(6)	11.27	279
(7)	12.45	172

**Table 4:** COD value measurement results for the mixture of rhodamine B-methyl orange 150 mg/L before and after the Fenton process.

Sample	COD (mg O_2_/g)	Decrease (%)
Mixture before catalytic	92.76	-
ZVI 1:1	7.32	92.11
Iron(II) sulfate	48.83	47.36

## Data Availability

Not applicable.

## Supplementary Materials

The following supporting information can be downloaded at: https://www.mdpi.com/article/10.3390/ma15010332/s1, Figure S1: Coordinates of tea leaf sampling location; Figure S2: (a) The curve of absorption wavelength of gallic acid; and (b) The curve of optimum incubation time for gallic acid at 698 nm; Figure S3: The curve of the gallic acid calibration standard; Figure S4: (a) Color of 0.1 M iron(II) sulfate solution; (b) Tea extract heated at 90 °C for 80 min; (c) synthesized nZVI before evaporation; (d) nZVI solid 1:1, (e) 1:2, and (f) 1:3; Figure S5: Absorbance of ZVI catalytic test for 50 mg/L ZVI concentration; Figure S6: Absorbance of ZVI catalytic test for 100 mg/L ZVI concentration; Figure S7: Absorbance of ZVI catalytic test for 150 mg/L ZVI concentration; Figure S8: Absorbance of iron(II) sulfate catalytic test for 150 mg/L iron(II) concentration.
