# Supplementary material for: Green Production of Zero-Valent Iron (ZVI) Using Tea-Leaf Extracts for Fenton Degradation of Mixed Rhodamine B and Methyl Orange Dyes
Source: Materials (Basel). 2022 Jan 3;15(1):332. doi: 10.3390/ma15010332 (PMC8746258; doi:10.3390/ma15010332)
Supplement: Supplementary file 1 [file materials-15-00332-s001.zip › materials-1533936-supplementary.pdf]

Supplementary Information

1. Tea Extract Preparation

The tea leaves (*Camellia sinensis*) for this study were taken on January 12, 2020, with an air temperature of 29°C, 11.00-12.00 AM in sunny weather conditions, from a local tea plantation in Citengah, Sumedang at an altitude of 1133 masl with coordinates 6°55'44.0"S 107°58 '28.3"E (Figure S1).

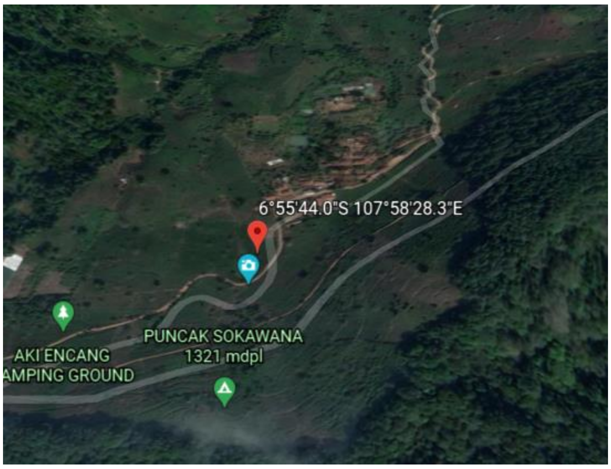

Figure S1. Coordinates of tea leaf sampling location.

2. Preparation of Gallic Acid Standard Curve and Determination of Polyphenol Content

Gallic acid is made in several standard variations, namely 1, 2, 3, 4, and 5 mg.L^{-1}. Then the gallic acid was reacted with 0.2 mL of Folin Ciocalteu reagent and 4 mL of 7.5% sodium carbonate, then incubated at room temperature. Then the standard was measured using a visible light spectrophotometer (HACH DR 3900, Japan). The experimental results showed that the maximum wavelength indicated by standard gallic acid was 698 nm (Figure S2(a)) with an optimal incubation time of 45 minutes (Figure S2(b)). Figure S3 shows the calibration curve for gallic acid.

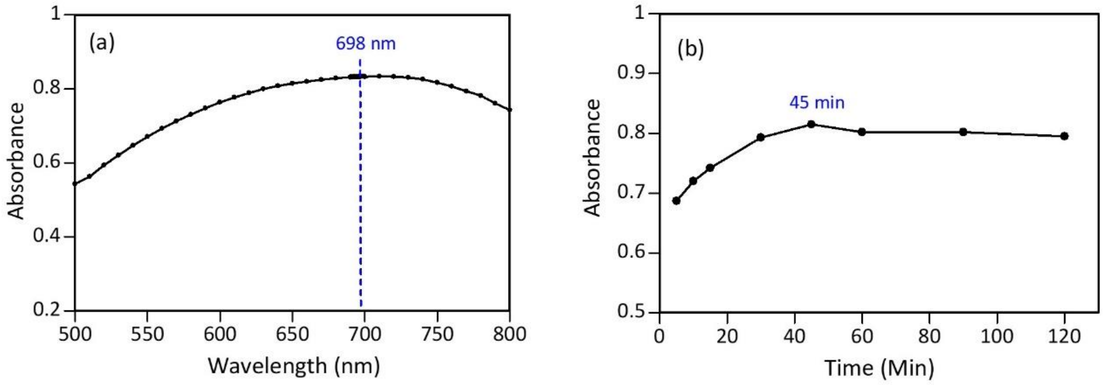

Figure S2. (**a**) The curve of absorption wavelength of gallic acid; and (**b**) The curve of optimum incubation time for gallic acid at 698 nm.

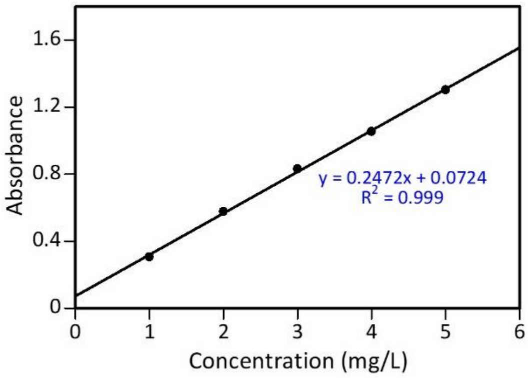

Figure S3. The curve of the gallic acid calibration standard.

3. Formation and Characterization of nZVI

The formation of nZVI was indicated by a change in the color of the iron(II) solution from clear greenish to blackish brown in Figure S4(a-c). From the synthesis process, nZVI solids were produced (Figure S5(d-f)) which visually did not show a significant difference at nZVI 1:1, 1:2, and 1:3.

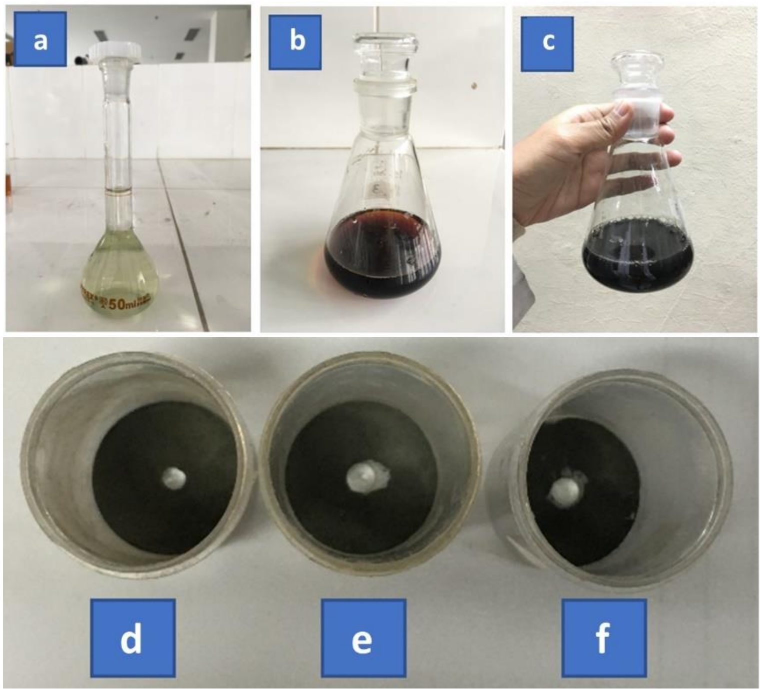

Figure S4. (**a**) Color of 0.1 M iron(II) sulfate solution; (**b**) Tea extract heated at 90°C for 80 minutes; (**c**) synthesized nZVI before evaporation; (**d**) nZVI solid 1:1, (**e**) 1:2, and (**f**) 1:3.

4. Color Intensity Test

An analysis was carried out to see any changes in the peak at a wavelength of 200-600 nm during the Fenton process. The measurement results showed that the mixed dyes that underwent a catalytic process using nZVI 1:1, 1:2, and 1:3 with concentrations of 50, 100, and 150 mg/L underwent the same

changes during the catalytic process from 30-180 minutes (Figgure S5-7). The peak at the wavelength of 506 nm did not shift and only experienced a decrease in color intensity, while the peak around the wavelength of 200-300 nm experienced a change. This peak is thought to originate from nZVI which has been converted to iron(II) during the Fenton process (Figure S8).

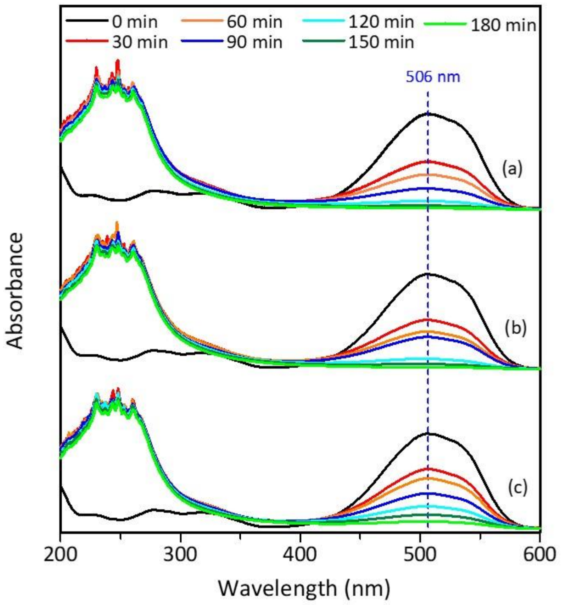

Figure S5. Absorbance of ZVI catalytic test for 50 mg/L ZVI concentration of: (**a**) ZVI 1:1; (**b**) ZVI 1:2; and (**c**) ZVI 1:3 at a mixture dye concentration of 50 mg/L.

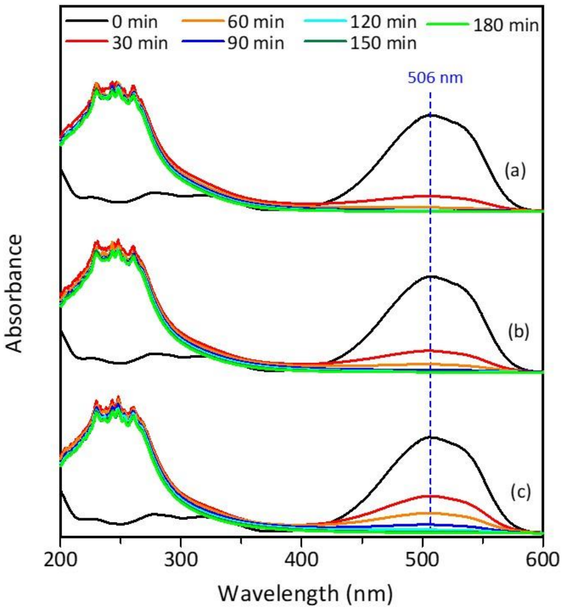

Figure S6. Absorbance of ZVI catalytic test for 100 mg/L ZVI concentration of: (**a**) ZVI 1:1; (**b**) ZVI 1:2; and (**c**) ZVI 1:3 at a mixture dye concentration of 50 mg/L.

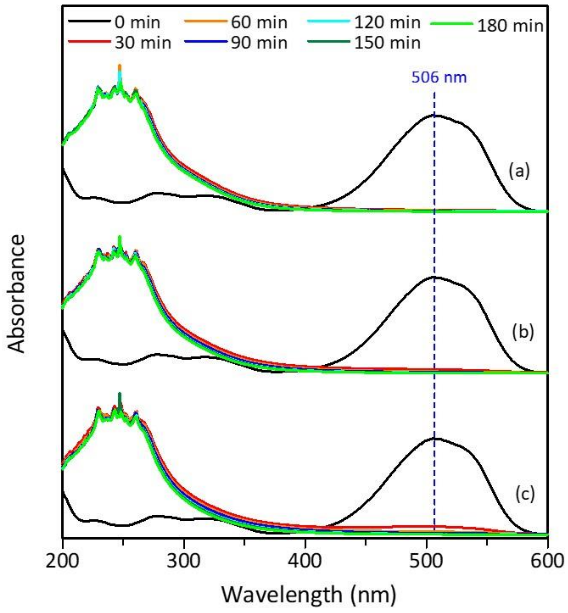

Figure S7. Absorbance of ZVI catalytic test for 150 mg/L ZVI concentration of: (**a**) ZVI 1:1; (**b**) ZVI 1:2; and (**c**) ZVI 1:3 at a mixture dye concentration of 50 mg/L.

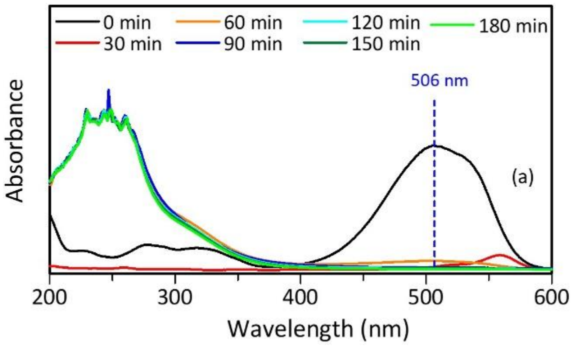

Figure S8. Absorbance of iron(II) sulfate catalytic test for 150 mg/L iron(II) concentration at a mixture dye concentration of 50 mg/L.
